# Tetramer organizing polyproline-rich peptides identified by mass spectrometry after release of the peptides from Hupresin-purified butyrylcholinesterase tetramers isolated from milk of domestic pig (*Sus scrofa*)

**DOI:** 10.1016/j.dib.2018.08.109

**Published:** 2018-08-31

**Authors:** Ashima Saxena, Tatyana Belinskaya, Lawrence M. Schopfer, Oksana Lockridge

**Affiliations:** a Division of Biochemistry, Walter Reed Army Institute of Research, 503 Robert Grant Avenue, Silver Spring, MD 20910-7500, USA; b Eppley Institute, University of Nebraska Medical Center, Omaha, NE 68198 USA; c Infectious Diseases Research Directorate, Naval Medical Research Center, 503 Robert Grant Avenue, Silver Spring, MD 20910-7500, USA

## Abstract

Milk of the domestic pig has 10 times more butyrylcholinesterase (BChE) per mL than porcine serum. We purified BChE from porcine milk by affinity chromatography on Hupresin-Sepharose. The pure porcine BChE (PoBChE) was a tetramer with a molecular weight of 340,000, similar to that of human BChE tetramers. The C-terminal 40 residues of PoBChE constitute the tetramerization domain. The glue that holds the 4 BChE subunits together is a polyproline-rich peptide. Mass spectrometry analysis of trypsin-digested PoBChE identified a variety of polyproline-rich peptides originating from 12 different proteins. The donor proteins exist in the nucleus or cytoplasm of cells and contribute their polyproline-rich peptides after a cell is degraded. The secreted PoBChE scavenges the polyproline-rich peptides and incorporates one polyproline peptide per PoBChE tetramer, where the polyproline peptide is bound noncovalently but very tightly with an estimated dissociation constant of 10^–12^ M. The most abundant polyproline-rich peptides were derived from acrosin, homeobox protein HoxB4, lysine-specific demethylase 6B, proline-rich protein 12, and proline-rich membrane anchor 1 (PRiMA). The research article associated with the data in this report can be found in Saxena et al. (2018). The Data in Brief report lists all the polyproline-rich peptides identified in PoBChE tetramers.

**Specifications table**

**Table:** 

Subject area	Biology
More specific subject area	Tetramer organizing polyproline-rich peptides of butyrylcholinesterase
Type of data	Amino acid sequences of polyproline-rich peptides released from pure PoBChE
How data was acquired	Liquid chromatography tandem mass spectrometry (LC–MS/MS) on the 6600 Triple-TOF mass spectrometer (AB Sciex).
Data format	Analyzed
Experimental factors	Pure PoBChE tetramers were deglycosylated with PNGaseF, denatured in a boiling water bath to release noncovalently bound polyproline-rich peptides, and digested with trypsin. Peptides were separated by Ultra High Performance Liquid Chromatography and analyzed by MS/MS. Peptide data were searched against the NCBInr 15Sep2014 database for *Sus scrofa* proteins using the Paragon algorithm from Protein Pilot v 5.01 (AB Sciex).
Experimental features	The amino acid sequence of the 574 amino acid PoBChE protein was determined by LC-MS/MS of trypsin-digested PoBChE.
Data source location	Omaha, Nebraska, USA
Data accessibility	The NCBI accession number for the PoBChE protein amino acid sequence is NP_001344438.1. https://www.ncbi.nlm.nih.gov/protein/NP_001344438.1
Related research article	Saxena A, Belinskaya T, Schopfer LM, Lockridge O. Characterization of butyrylcholinesterase from porcine milk. Arch Biochem Biophys 2018, 652:38-49

**Value of the data**

• The finding that PoBChE is abundant in porcine milk [1] and in human milk [2], [3] leads to new questions. What is the function of BChE in milk? Does the infant benefit from BChE in mother׳s milk as a consequence of the inactivation of octanoyl-ghrelin by BChE, thus reducing the infant׳s anxiety and stress [4]? Ghrelin has a role in regulation of neural circuits and body growth during neonatal development [5]. Is BChE in milk involved in these ghrelin-related effects?
• The set of polyproline-rich peptides in PoBChE tetramers is different from the set in human BChE tetramers [6], [7]. PoBChE was purified from milk. Human BChE was purified from plasma. Porcine plasma has a very low amount of BChE. Since incorporation of a polyproline-rich peptide stabilizes the BChE tetramer, is the limiting factor for the level of BChE the availability of polyproline-rich peptides? Does it mean the cells in the porcine mammary gland (source of milk BChE) undergo a higher rate of degradation and therefore produce more polyproline-rich peptides than cells in porcine liver (source of plasma BChE)?
• Are excess polyproline-rich peptides toxic to cells? Does BChE incorporate polyproline peptides from degraded cells because degraded cells are a convenient source? If excess polyproline-rich peptides are toxic to cells, BChE might be protecting the organism by scavenging these peptides.
• The BChE tetramer incorporates not only short polyproline-rich peptides, but also long proteins that contain a polyproline-rich region. An example is the C5 variant of human BChE whose tetrameric structure includes a 60 kDa lamellipodin protein [8]. Does the BChE tetramer, and by implication the AChE tetramer, serve as a carrier of proteins that confer the observed non-cholinergic functions of BChE and AChE in bone development [9]? Examples of polyproline-rich donor proteins that could account for non-cholinergic functions of BChE and AChE include the Homeobox protein Hox-B4 which is a transcription factor involved in development, and the Formin-homology domain containing protein 1 which is involved in cell migration and adhesion.
• The ability of BChE subunits to assemble into stable, long-lived tetramers by binding the polyproline-rich region of a protein, suggests that BChE could serve as a delivery vehicle for any protein that has been engineered to include a polyproline-rich tag.

## 1 Data overview

We present all the polyproline-rich peptides associated with PoBChE tetramers that were identified by mass spectrometry. In addition we show the complete amino acid sequence of the full-length protein that donated each polyproline-rich peptide, the location of the polyproline-rich peptide within the full-length protein sequence, and the abundance of the polyproline-rich peptide relative to PoBChE peptides. A brief description is given of the function of the donor protein and its location within a cell.

## 2 Experimental design, materials, and methods

### 2.1 Purification of PoBChE

PoBChE was purified from defatted porcine milk (1200 mL) by affinity chromatography on procainamide-Sepharose [10]. A side fraction was purified to homogeneity on 16 mL of Hupresin-Sepharose (CHEMFORASE, Mont-Saint-Aignan, France). Contaminating proteins were washed off with 20 mM Tris.HCl pH 7.5, 0.05% azide followed by 0.3 M NaCl in 20 mM Tris.HCl pH 7.5, 0.05% azide. PoBChE was eluted with 0.1 M tetramethylammonium bromide in 20 mM Tris.HCl pH 7.5, 0.05% azide at room temperature.

### 2.2 Sample preparation for LC–MS/MS

The Hupresin-purified PoBChE was reduced in volume from 7.3 mL to 0.17 mL in a Centricon YM-30 spin filter. After the buffer was changed to 10 mM ammonium bicarbonate pH 8, the PoBChE was deglycosylated with 1 µl of PNGaseF for 1 h. Noncovalently bound polyproline-rich peptides were released from PoBChE by denaturing the protein in a boiling water bath for 5 min. The denatured 170 µg of PoBChE protein in 170 µL of 10 mM ammonium bicarbonate pH 8 was digested with 2 µg of trypsin (Promega V511C, 2 µg in 5 µL) for 20 h at 37 °C in a humidified chamber. Particles that could clog the small diameter tubing in the Ultra High Pressure Liquid Chromatography column were removed by centrifuging the digest for 30 min at 14,000 rpm in a microfuge. A 10 µL aliquot from the top of the centrifuged digest was transferred to an autosampler vial. The protein concentration in the digest was estimated at 1 µg/µL.

### 2.3 LC–MS/MS

The protocol for liquid chromatography tandem mass spectrometry (LC–MS/MS) is described in detail in [11]. In brief, peptides in a 5 µL volume were separated on a cHiPLC Nanoflex microchip column (Eksigent, Dublin, CA) packed with ChromXP C18. The eluted peptides were electrosprayed into the 6600 Triple-TOF mass spectrometer (AB Sciex) where mass spectra were collected in positive mode. Peptides were fragmented by collision-induced dissociation with nitrogen gas. The Triple-TOF data were searched against the NCBInr 15Sep2014 database for *Sus Scrofa* proteins using the Paragon algorithm from Protein Pilot (AB Sciex) (Table 1, Table 2, Table 3, Table 4, Table 5, Table 6, Table 7, Table 8, Table 9, Table 10, Table 11, Table 12).

**Table 1:** Acrosin peptides in PoBChE tetramers.

Name	Gene	Gi #	peptide	length	count
Acrosin	ACR	47522886	APPPPPPPPPPP	12	12
		P08001	PAPPPPPPPPPP	12	12
			PPAPPPPPPPPP	12	6
			PPAPPPPPPPPPPPPP	16	3
			PPPAPPPPPPPPPPPP	16	9
			APPPPPPPPPPPPPPP	16	3
			PAPPPPPPPPPPPPPP	16	3
			PPPPPPPPPPPPPPPQQ	17	4
			APPPAPPPPPPPPP	14	2
			PAPPPAPPPPPPPP	14	2
			PPPAPPPPPPPP	12	7
			APPPPPPPPPPPPP	14	1
			PAPPPPPPPPPPPP	14	2
			PPPAPPPPPPPPPP	14	2
			APPPPPPPPPPPP	13	2
			PAPPPPPPPPPPP	13	2
			PPPAPPPPPPP	11	1
			PPPPPPPPPPPP	12	5
			PPPPPPPPPPPPP	13	11
			PPPPPPPPPPPPPP	14	7
			PPPPPPPPPPPPPPP	15	1
			PPPPPPPPPPPPPPPPP	17	20
			PPPPPPPPPPPPPPPPPP	18	13
			PPPPPPPPPPPPPPPPPPP	19	6
			PPPPPPPPPPPPPPPPQQ	18	2

Total peptide count 138.

138 peptides for acrosin compared to 10978 peptides for PoBChE calculates to 1.25%.

**Table 2:** Homeobox protein Hox-B peptides in PoBChE tetramers.

Name	Gene	Gi #		Length	count
Homeobox protein Hox-B4	HOXB4	311267494	RDPGPPPPPPPPPPPPPPPGL	21	32
			DPGPPPPPPPPPPPPPPPGL	20	9
			GPPPPPPPPPPPPPPPGL	18	10
			PGPPPPPPPPPPPPPPPGL	19	24
			GPPPPPPPPPPPP	13	8
			PGPPPPPPPPPPP	13	8
			PGPPPPPPPPPPPPPPP	17	1
			PPPPPPPPPPPP	12	5
			PPPPPPPPPPPPP	13	11
			PPPPPPPPPPPPPP	14	7
			PPPPPPPPPPPPPPP	15	1

Total peptide count 116.

116 homeobox peptides/ 10978 PoBChE peptides = 1.0%.

**Table 3:** Lysine – specific demethylase 6B peptides in PoBChE tetramers.

Name	Gene	Gi #	peptide	Length	count
Lysine-specific demethylase 6B	KDM6B	545860221	PPPPPPPPPPPPPPPLPGLAT	21	1
			PPPPLPPPPPPPPPPPP	17	1
			LPPPPPPPPPPP	12	17
			LPPPPPPPPPPPP	13	17
			LPPPPPPPPPPPPPPP	16	5
			PLPPPPPPPPPP	12	16
			PLPPPPPPPPPPP	13	15
			PLPPPPPPPPPPPPPP	16	4
			PLPPPPPPPPPPPPPPP	17	1
			PPLPPPPPPPPP	12	7
			PPLPPPPPPPPPP	13	9
			PPLPPPPPPPPPPPPP	16	4
			PPPLPPPPPPPP	12	21
			PPPLPPPPPPPPP	13	19
			PPPLPPPPPPPPPPPP	16	9
			PPPPLPPPPPPP	12	6
			PPPPLPPPPPPPP	13	26
			PPPPLPPPPPPPPPPP	16	7
			PPPPPPPPPPPP	12	5
			PPPPPPPPPPPPP	13	11
			PPPPPPPPPPPPPLPGL	17	1
			PPPPPPPPPPPPPP	14	7
			PPPPPPPPPPPPPPP	15	1

Total peptide count 210.

210 Lysine-specific demethylase 6B peptides/10978 PoBChE peptides = 1.9%.

**Table 4:** Zinc finger homeobox protein 4 peptides in PoBChE tetramers.

Name	Gene	Gi #	peptide	Length	Count
Zinc finger homeobox protein 4	ZFHX4	545821241	TPPPPPPPPPPPPPPPPPPPSA	22	2
			TPPPPPPPPPPPPPPSSL	18	1
			PPPPPPPPPPPP	12	10
			PPPPPPPPPPPPP	13	11
			PPPPPPPPPPPPPP	14	7
			PPPPPPPPPPPPPPP	15	1
			PPPPPPPPPPPPPPPPP	17	20
			PPPPPPPPPPPPPPPPPP	18	13
			PPPPPPPPPPPPPPPPPPP	19	6

Total peptide count 71.

69 Zinc finger homeobox protein 4 peptides/10978 PoBChE peptides = 0.6%.

**Table 5:** Zinc finger CCCH domain-containing protein 4 peptides in PoBChE tetramers.

Name	Gene	Gi #	peptide	Length	Count
Zinc finger CCCH domain-containing protein 4	ZC3H4	545831605	GGPPPPPPPPPPPPGPPQM	19	19
			GPPPPPPPPPPPPGPPQM	18	1
			GPPPPPPPPPPP	12	8
			PPPPPPPPPPPP	12	5

Total peptide count 33.

33 Zinc finger CCCH domain-containing protein 4 peptides/10978 PoBChE peptides = 0.3%.

**Table 6:** Disabled homolog 2-interacting protein-like isoform 1 peptides in PoBChE tetramers.

Name	Gene	Gi #	peptide	Length	Count
Disabled homolog 2-inter acting protein-like isoform 1	DAB2IP	545804284	IDQPPPPPPPPPPAPR	16	12

Total peptide count 12.

12 Disabled homolog 2-interacting protein-like isoform 1 peptides/10987 PoBChE peptides = 0.1%.

**Table 7:** Protein FAM171A2 peptides in PoBChE tetramers.

Name	Gene	Gi #	peptide	Length	Count
Protein FAM171A2	FAM171A2	545857706	AAAPPPPPPPPPPAPPR	17	4

Total peptide count 4.

4 Protein FAM171A2 peptides/10987 PoBChE peptides = 0.03%.

**Table 8:** FH2 domain-containing protein 1 peptides in PoBChE tetramers.

Name	Gene	Gi #	peptide	Length	Count
FH2 domain-containing protein 1	FHDC1	545845605	PPPPSPPPPPPPP	13	1
			PPPPSPPPPPPP	12	4
			PPPSPPPPPPPP	12	4
			PPSPPPPPPPPP	12	1

Total peptide count 10.

10 FH2 domain-containing protein 1 peptides/10987 PoBChE peptides = 0.09%.

**Table 9:** Proline-rich protein 12 peptides in PoBChE tetramers.

Name	Gene	Gi #	peptide	Length	Count
Proline-rich protein 12	PRR12	335290066	APPPPPPPPPPPPPASEPK	19	2
			APPPPPPPPPPP	12	12
			APPPPPPPPPPPP	13	2
			APPPPPPPPPPPPP	14	2
			LPPPPPPPPPPP	12	17
			LPPPPPPPPPPPP	13	17
			LPPPPPPPPPPPPPPP	16	5
			LPPPPPPPPPPPPPPPP	17	1
			PPPPPPPPPPPP	12	5
			PPPPPPPPPPPPP	13	11
			PPPPPPPPPPPPPP	14	7
			PPPPPPPPPPPPPPP	15	1
			PPPPPPPPPPPPPPPPP	17	21
			PPPPPPPPPPPPPPPPPP	18	14
			PPPPPPPPPPPPPPPPPPPP	19	6

Total peptide count 123.

123 Proline-rich protein 12 peptides/10987 PoBChE peptides = 1.1%.

**Table 10:** WAS/WASL-interacting protein family member 2 isoform X1 peptide in PoBChE.

Name	Gene	Gi #	peptide	Length	Count
WAS/WASL-interacting protein family member 2 isoform X1	WIPF2	346716187	PIPPPPPPPPGPPPPPTF	18	5
			MPIPPPPPPPPGPPPPPTF	19	1

Total peptide count 6.

6 WAS/WASL-interacting protein family member 2 isoform X1 peptides/10987 PoBChE peptides = 0.05%.

**Table 11:** Proline-rich protein 16 peptide in PoBChE tetramers.

Name	Gene	Gi #	peptide	Length	Count
Proline-rich protein 16	PRR16	545812042	PNPPPPPPR	9	1

Total peptide count 1.

1 Proline-rich protein 16 peptide/10987 PoBChE peptides = 0.009%.

**Table 12:** Proline-rich membrane anchor 1 peptides in PoBChE tetramers.

Name	Gene	Gi #	peptide	Length	Count
Proline-rich membrane anchor 1	PRIMA1	350587156	PPLPPPPPPPPPPR	14	3
			PLPPPPPPPPPP	12	23
			PLPPPPPPPPPPP	13	9
			PPPLPPPPPPPP	12	21
			PPPLPPPPPPPPP	13	19
			PPPPLPPPPPPP	12	6
			PPPPLPPPPPPPP	13	26

Total peptide count 107.

107 Proline-rich membrane anchor 1 peptides/10987 PoBChE peptides = 1%.

>sp|P08001|ACRO_PIG Acrosin OS=Sus scrofa GN=ACR PE=1 SV=5 MLPTAVLLVLAVSVAARDNATCDGPCGLRFRQKLESGMRVVGGMSAEPGAWPWMVSLQIFMYHNNRRYHTCGGILLNSHWVLTAAHCFKNKKKVTDWRLIFGANEVVWGSNKPVKPPLQERFVEEIIIHEKYVSGLEINDIALIKITPPVPCGPFIGPGCLPQFKAGPPRAPQTCWVTGWGYLKEKGPRTSPTLQEARVALIDLELCNSTRWYNGRIRSTNVCAGYPRGKIDTCQGDSGGPLMCRDRAENTFVVVGITSWGVGCARAKRPGVYTSTWPYLNWIASKIGSNALQMVQLGTPPRPSTPAPPVRPPSVQTPVRPPWYFQRPPGPSQQPGSRPRPVSAKPPQALSFAKRLQQLIEALKGTAFSSGRSYYETETTDLQELPAS.

27 residues PAPPPAPPPPPPPPPPPPPPPPPPPQQ MW 2648.4.

Acrosin is the major proteinase present in the acrosome of mature spermatozoa. It is a typical serine proteinase with trypsin-like specificity. It is stored in the acrosome in its precursor form, proacrosin. The active enzyme functions in the lysis of the zona pellucida, thus facilitating penetration of the sperm through the innermost glycoprotein layers of the ovum. The mRNA for proacrosin is synthesized only in the postmeiotic stages of spermatogenesis. In humans proacrosin first appears in the haploid spermatids. https://www.gtexportal.org/home/gene/ACR The human acrosin gene is expressed in testis and to a small extent in mammary breast tissue, lung, spleen, adipose tissue, and tibial artery.

>XP_003131596.1 PREDICTED: homeobox protein Hox-B4 [Sus scrofa] MAMSSFLINSNYVDPKFPPCEEYSQSDYLPSDHSPGYYAGGQRRESSFQPEAGFGRRAACTVQRYAACSPRAPAPPPSGALLPEPGQRCEAVSSSPPPPPCAQNPLHPSPSHSACKEPVVYPWMRKVHVSTVNPNYAGGEPKRSRTAYTRQQVLELEKEFHYNRYLTRRRRVEIAHALCLSERQIKIWFQNRRMKWKKDHKLPNTKIRSGGPASAAGGPPGRPNGGPPAL.

21 residues RDPGPPPPPPPPPPPPPPPGL MW 2069.1.

The HOXB4 gene is a member of the Antp homeobox family and encodes a nuclear protein with a homeobox DNA-binding domain. It is included in a cluster of homeobox B genes located on human chromosome 17. The encoded protein functions as a sequence-specific transcription factor that is involved in development. Intracellular or ectopic expression of this protein expands hematopoietic stem and progenitor cells in vivo and in vitro, making it a potential candidate for therapeutic stem cell expansion.

>XP_005657086.1 PREDICTED: LOW QUALITY PROTEIN: lysine-specific demethylase 6B [Sus scrofa] MHRAVDPPGARTAREAFALGGLSCAGAWSSCPPHPPPRSAWLPGGRCSASIGQPPLSAPLPPSHGSSSGHPNKLYFAPGTPNPRPLHGKLESLHGCVQALLREPAQPGLWEQLGQLYESEHDSEEAIRCYHSALRYGGSLAELGPRIGRLQQAQLWNFHAGSCQHRPKVLPPLEQVWNLLHLEHKRNYGAKRGGPPVKRAAEPPVVQPVPPAALSGPSGEEGLSPGGKRRRGCNSEQTGLPPGLSPPFQLTKPGLWSTLHGDAWGPERKGTAPPERQEQRHSLPRHPYPYPAPAYPSRTPWPRLVPAAPPGPGPXPPGAESHGCPPATRPPGSDLRESRVQRSRMDSSVSPAATTACVPYAPSRPPALPGTTTSSSSSSSNTGLRGVEPSPGIPGADHYQTPALEVSSHQGRLGPSAHSSRKPFLAAPAATPHLSLPPGPPSPPPPPCPRLLRPPPPPAWLKGPACRAAREDGEILEELFFGAEGRPRPPPPPLPHREGFLGPPAPRFSVGTQDSHTPPTPPTTSSSSSNNGSHSSSPTGSVSFPPPPYLARSMDPLPRPPSPTLSPQDPPLAPLSLALPPAPPSSCHQNTSGSFRRPESPRPRVSFPKTPEVGPGPSPGPLNKAPQPVPSRVGELPARGPRLFDFPPTPLEDQFEEPAEFKILPDGLANIMKMLDESIRKEEEQQQQEAGVVPPPPLKEPFASLQPPFPTDTAPATTAATTAATTTATQEEEKKPPPALPPPPPLAKFPPPPQPQPPPPPLPPPASPASLLKSLASVLEGQKYCYRGTGAAVATRPGPLPTTQYSPGPPSGATAPPPTSAAPSAQGSPQPSASSSSQFSTSGGPWARERRAGEEPAPGPTTPAPPPPPLPLPPARSESEVLEEISRACETLVERVGRGATDPADPADTADPVDTGAERLLPPAQAKEEAGGASAVAAAAAGPGSSKRRQKEHQKEHRRHRRACKDSVGRRPREGRAKAKAKAPKEKSRRVLGNLDLQSEEIQGREKARPDLGGASKAKPPTAPAPLPAPAPSTQSTPPSAPVPGKKAREEAPGPPGVSRADMLKLRSLSEGPPKELKIRLIKVESGDKETFIASEVEERRLRMADLTISHCAADVVRASKNAKVKGKFRESYLSPAQSVKPKINTEEKLPREKLNPPTPSIYLESKRDAFSPVLLQFCTDPRNPITVIRGLAGSLRLNLGLFSTKTLVEASGEHTVEVRTQVQQPSDENWDLTGTRQIWPCESSRSHTTIAKYAQYQASSFQESLQEEKESEDEESEEPDSTTETPPSSAPDPKNHHIIKFGTNIDLSDAKRWKPQLQELLKLPAFMRVTSTGNMLSHVGHTILGMNTVQLYMKVPGSRTPGHQENNNFCSVNINIGPGDCEWFAVHEHYWETISAFCDRHGVDYLTGSWWPILDDLYASNIPVYRFVQRPGDLVWINAGTVHWVQATGWCNNIAWNVGPLTAYQYQLALERYEWNEVKNVKSIVPMIHVSWNVARTVKISDPDLFKMIKFCLLQSMKHCQVQRESLVRAGKKIAYQGRVKDEPAYYCNECDVEVFNILFVTSENGSRNTYLVHCEACARRRSAGLQGVVVLEQYRTEELAQAYDAFTLAPASTSR.

28 residues PLPPPPLPPPPPPPPPPPPPPPLPGLAT MW 2737.5.

Histone demethylase specifically demethylates ׳Lys-27׳ of histone H3, thereby playing a central role in histone code (PubMed:17825402, PubMed:17851529, PubMed:17713478, PubMed:18003914). Demethylates trimethylated and dimethylated H3 ׳Lys-27׳ (PubMed:17825402, PubMed:17851529, PubMed:17713478, PubMed:18003914). Plays a central role in regulation of posterior development, by regulating HOX gene expression (PubMed:17851529). Involved in inflammatory response by participating in macrophage differentiation in case of inflammation by regulating gene expression and macrophage differentiation (PubMed:17825402). Plays a demethylase-independent role in chromatin remodeling to regulate T-box family member-dependent gene expression by acting as a link between T-box factors and the SMARCA4-containing SWI/SNF remodeling complex.

>XP_005663076.1 PREDICTED: LOW QUALITY PROTEIN: zinc finger homeobox protein 4 [Sus scrofa] METCDSPPISRQENGQSTSKLCGTAQLDNEVPEKVAGMEPDRENSSTDDNLKTDERKSEVLLGFSVENAAATQVTSAKEIPCNECATSFPSLQKYMEHHCPNARLPVLKDDNESEISELEDSDVENLTGEIVYQPDGSAYIIEDSKESGQNAQTGANSKLFSTAMFLDSLASAGEKSDQSASAPMSFYPQIINTFHIASSLGKPFTADQAFPNTSALAGVGPVLHSFRVYDLRHKREKDYLTSDGSAKNSCVSKDVPNNVDLSKFDGCVSDGKRKPVLMCFLCKLSFGYIRSFVTHAVHDHRMTLNEEEQKLLSNKCVSAIIQGIGKDKEPLISFLEPKKSTSVYPHFSTTNLIGPDPTFRGLWSAFHVENGDSLPAGFAFLKGSAGTSGSAEQPLGITQMPKAEVTLGGLSSLVVNTPITSVSLSNASSESSKMSESKDQENDCERPKESNALHPNGECPVKSEPTEAGDEDEEDAYSNELEDEEVLGELTDSIGNKDFPLLNQSISPLSSSVLKFIEKGPSSSSASVTDDAEKKKPTAAVRASGGVANSYGIGGKDFAEASASKDGATAAHSSEPARGDEDSSATPHQHGFTPSAPGTPGPGGDGSPGSGIECPKCDTVLGSSRSLGGHMTMMHSRNSCKTLKCPKCNWHYKYQQTLEAHMKEKHPEPGGSCVYCKTGQPHPRLARGESYTCGYKPFRCEVCNYSTTTKGNLSIHMQSDKHLNNVQNLQNGNGEQVFGHSAPAPNTSLSGCGTPSPSKPKQKPTWRCEVCDYETNVARNLRIHMTSEKHMHNMMLLQQNMKQIQHNLHLGLAPAEAELYQYYLAQNIGLTGMKLENPGDPQLMLNPFQLDPATAAALAPGLVNNELPPEIRLASGQLMGDDLSLLTAGELSPYISDPALKLFQCAVCNKFTSDSLEALSVHVSSERSLPEEEWRAVIGDIYQCKLCNYNTQLKANFQLHCKTDKHMQKYQLVAHIKEGGKSNEWRLKCIAIGNPVHLKCNACDYYTNSVDKLRLHTTNHRHEAALKLYKHLQKQEGAVNPESCYYYCAVCDYSTKVKLNLVQHVRSVKHQQTEGLRKLQLHQQGLAPEEDNLSEIFFVKDCPPNELETASLGARTCEDDLLEQQLRAPSEEQSEETEGASRPTAVAEDDEKDTSERDNNEGKNSNKDTGIITPEKELKVSVAGGTQPLLLAKEEDVATKRSKPTEDSKFCHEQFYQCPYCNYNSRDQSRIQMHVLSQHSVQPVICCPLCQDVLSNKMHLQLHLTHLHSVSPDCVEKLLMTVPVPDVMMPNSLLLPAAASEKSERDTPAAITAEGPGKYSGESPMDDKSMAGLDDSKAIMEIKSEEQKPTKEPTEASEWNKNSSKDGKISDPLQDQLSEQQKRQPLSVSDRHVYKYRCNHCSLAFKTMQKLQIHSQYHAIRAATMCNLCQRSFRTFQALKKHLEAGHPELSEAELQQLYASLPVNGELWAESETMAQDDHALEQEMEREYEVDHEGKASPVGSDSSSIPDDMGSEPKRTLPFRKGPNFTMEKFLDPSRPYKCTVCKESFTQKNILLVHYNSVSHLHKLKKVLQEASSPVPQETNSSTDNKPYKCSICNVAYSQSSTLEIHMRSVLHQTKARAAKLEPSSHVVSGHSAANVSSPGQGMLDSMSLAGVSSKDTHLDAKELNKKQTPELISAQPAHHPPQSPAQIQMQLQHELQQQAAFFQPQFLNPAFLPHFPMTPEALLQFQQPQFLFPFYIPGTEFSLGPDLGLPGSAAFGMPGMTGMAGSLLEDLKQQIQTQHHVGQTQLQILQQQAQQYQATQPQLQSQKPQQQPQPQPQQQQASKLLKQEQTTLASAECPIVKDIPSFKEAEEMAKKQDKPKQEVXSEGEGLKEGKDEKKQKSSEPSILPPRIASGARGNAAKALLENFGFELVIQYNENRQKVQKKGKSGEGESTEKLECGTCGKLFSNVLILKSHQEHVHGQFFPYGALEKFARQYREAYDKLYPISPSSPETPPPPPPPPPLPPAPPQPASLGPVKLPSTVSTPIQAPPPPPRVQLPVSLDLPLFPSIMMQPVQHPALPPQLALQLPQMDTLSADLTQLCQQQLGLDPNFLRHSQFKRPRTRITDDQLKILRAYFDINNSPSEEQIQEMAEKSGLSQKVIKHWFRNTLFKERQRNKDSPYNFSNPPITVLEDIRIDPQPSSLEHYKSDASFSKRSSRTRFTDYQLRVLQDFFDTNAYPKDDEIEQLSTVLNLPTRVIVVWFQNARQKARKSYENQAEAKDNEKRELTNERYIRTSNMQYQCKKCNVVFPRIFDLITHQKKQCYKDEDDDAQDESQTEDSMDATDQVVYKHCTVSGQTEAAKNAPVAAASSGSGASTPLLPSPKPEPEKTSPKPEYPTEKPKQSDPSPPSQGTKPALPLASTSSEPPQAAAAQPQPQPPKQPQLIGRPPSASQTPIPSSPLQISMTSLQNSLPPQLLQYQCDQCTVAFPTLELWQEHQHMHFLAAQNQFLHSPFLERPMDMPYMIFDPNNPLMTGQLLSGFLTQMPPQNASSQTPASATVAASLKGNWDDKEDTNCSEKEGGNSGEDQHRDKRXRTTITPDKLEILYEKYLLDSNPTRKMLDHIAREVGLKKRVVQVWFQNTRARERKGQFRAVGPAQSHKRCPFCRALFKAKSALESHIRSRHWNEGKQAGYSLPPSPLIATEDGGESPQKYIYFDYPSLPLTKIDLSSENELASTVSTPVSKTAELSPKNLLSPSSFKAECSEDVENLNAPPAEAGYDQNKPDFDETSSINTAISDATTGDEGNAEMESTTGSSGDVKPALSPKEPKTLDTLAKTATTPTTEVCDEKFLFSLTSPSIPFNDKDGDHDQSFYITDDPDDNADRSETSSIADPSSPNPFGSSNPFKSKSNDRPGHKRFRTQMSNLQLKVLKACFSDYRTPTMQECEMLGNEIGLPKRVVQVWFQNARAKEKKFKINIGKPFMINQSGTEGTKPECTLCGVKYSARLSIRDHIFSKQHISKVRETVGSQLDREKDYLAPTTVRQLMAQQELDRIKKASDVLGLAVQQPSMMDSSSLHGISLPAAYPGLPGLPPVLLPGMNGPSSLPGFPQNSNTLTPPGAGMLGFPTSATSSPALSLSSAPTKPLLQSGQQTEPQNKESEKKQTKPNKVKKIKEEELEATKPEKHPKKEEKISSALSVLGKVVGETHVDPSQLQALQNAIAGDPASFLGGQFLPYFIPGFASYFTPQLPGTVQGGYLPPVCGMESLFPYGPTMPQTLAGLSPGALLQQYQQYQQNLQDSLQKQQKQQQQEQPQKPGQAKTSKGESEPPQNASDASETKEDKSTATESTKEEPQLESKSADFSDTYVVPFVKYEFICRKCQMMFTDEDAAVNHQKSFCYFGQPLIDPQETVLRVPVSRYQCLACDVAISGNEALSQHLQSSLHKEKTIKQAMRNAKEHVRLLPHSVCSPNPNTTSTSQSAASSNTYPHLSCFSMKSWPNILFQASARRAASSPSSPPSLSLPSTVTSSLCSTSGVQTSLPTESCSDESDSELSQKLEDLDNSLEVKAKPASGLDGNFNSIRMDMFSV.

22 residues TPPPPPPPPPPPPPPPPPPPSA MW 2121.1.

18 residues TPPPPPPPPPPPPPPSSL MW 1764.9.

May play a role in neural and muscle differentiation. May be involved in transcriptional regulation.

>XP_005664683.1 PREDICTED: LOW QUALITY PROTEIN: zinc finger CCCH domain-containing protein 4 [Sus scrofa] MEAAPGTPPPPPSESPPPPSPPLPSTPSPPPCSPDACPATPHLLHHRLPLPDDREDGELEEGELEDDGAEETQDTSGGPERSRKEKGDKHHSDSDEEKSHRRLKRKRKKEREKEKRRSKKRRKSKHKRHASSSDDFSDFSDDSDFSPSEKGHRKYREYSPPYAPSHQQYPPSHTTPLPKKAYSKMDSKGYSMYEDYENEQYGEYEGDEEEDMGKDDYDDFTKELNQYRRAKEGSSRGRGSRGRGRGYRGRGSRGGSRGRGMGRGSRGRGRGSMGGDHPEDDEDFYEEEMEYGESEEPMGDEDYDDYSKELNQYRRSKDGRGRGLSRGRGRGSRGRGKGMGRGRGRGGSRGGMNKGGMNEDDDFYDEDMGDGGGGGGSYRRSDHDKPHQQSDKKGKVICKYFVEGRCTWGDHCNFSHDIELPKKRELCKFYITGFCARAENCPYMHGDFPCKLYHTTGNCINGDDCMFSHDPLTEETRELLDKMLADDAEAGAEDEKEVEELKKQGINPLPKPPPGVGLLPTPPRPPGPPAPTSPNGRPLQPMPVHEPLSPQQLQQQQDMYNKKIPSLFEIVVRPTGQLAEKLGVRFPGPGGPPGPMGPGPNMGPPGPMGGPMHPDMHPDMHPDMHPDMHPDMPMGPGMNPGPPMGPGGPPMMPYGPGDSPHSGMMPPIPPAQNFYENFYQQQEGMEMEPGLIGDTEDYGHYEELPGEPGEHLFPEHPLEPDSFSEGGPPGRPKPGAGVPDFLPSAQRALYLRIQQKQQEEEERARRLAESSKQDRENEEGDPGNWYSSDEDEGGSSVTSILKTLRQQTSSRPQASGGELSSSGLGDPRLQKGHPTGGRLADPRLSRDPRLSRHAEASGGSGPGDTGPSDPRLARSLPTPRPKGGLHSSPGGPSGSKGSGPPPAEEEEGERALREKAVNIPLDPLPGHPLRDPRSQLQQFSHIKKDVTLSKPSFARTVLWNPEDLIPLPIPKQDAVPPVPVALQSMPALDPRLHRTTTTSGPPNPRQRPGTSTDPSASGSNLPDFELLSRILKTVNATGPSAAPGPGDKPSDPRVRKTPTDPRLQKPADSATSSRAAKPGSTEVSPSASPSGESSPPATAPYDPRVLAAGGLGQGSGSGQSSVLSGISLYDPRTPNAGGKATEPAADTGTQPKGPEGNGKSAATKAKEPPFVRKSALEQPESGKPGADGGAAAATDRYNSYNRPRPKATPAAAASGTPPPEGASPQPGVHNLPVPTLFGTVKQAPKTGSGSPFAGNSPAREGEQDAGSLKDVFKGFDPTASPFCQ.

19 residues GGPPPPPPPPPPPPGPPQM MW 1806.9.

NCBI and UniProt have ZC3H4 zinc finger CCCH-type containing protein 4 (Homo sapiens).

This gene encodes a member of a family of CCCH (C-x8-C-x5-C-x3-H type) zinc finger domain-containing proteins. These zinc finger domains, which coordinate zinc finger binding and are characterized by three cysteine residues and one histidine residue, are nucleic acid-binding. Other family members are known to function in post-transcriptional regulation.

>XP_003353684.3 PREDICTED: LOW QUALITY PROTEIN: disabled homolog 2-interacting protein-like isoform 1 [Sus scrofa] MSAGGSARKSTGRPSYYYRLLRRPRLQRQRSRSRSRTRPARESPQERPGSRRSLPGSLSEKSPSMEPSAATPFRVTGFLSRRLKGSIKRTKSQPKLDRNHSFRHILPGFRSAAAAAAAASAADNERSHLMPRLKESRSHESLLSPSSAVEALALSMEEEVVIKPVHSSILGQDYCFEVTTSSGSKCFSCRSAAERDKWMENLRRAVHPNKDNSRRVEHILKLWVIEAKDLPAKKKYLCELCLDDVLYARTTGKLKTDNVFWGEHFEFHNLPPLRTVTVHLYRETDKKKKKERNSYLGLVSLPAASVAGRQFVEKWYPVVTPNPKGGKGPGPMIRIKARYQTITILPMEMYKEFAEHITNHYLGLCAALEPILSAKTKEEMASALVHILQSTGKVKDFLTDLMMSEVDRCGENEHLIFRENTLATKAIEEYLKLVGQKYLQDALGEFIKALYESDENCEVDPSKCSAADLPEHQGNLKMCCELAFCKIINSYCVFPRELKEVFASWRQECSSRGRPDISERLISASLFLRFLCPAIMSPSLFHLLQEYPDDRTARTLTLIAKVTQNLANFAKFGSKEEYMSFMNQFLEHEWTNMQRFLLEISNPETISNTAGFEGYIDLGRELSSLHSLLWEAVSQLEQSIVSKLGPLPRILRDVHTALSTPGSGQLTGTNDLASTPGSGSSSISAGLQKMVIENDLSGLIDFTRLPSPTPENKDLFFVTRSSGVQPSPARSSSYSEANEPDLQMANGGKSLSMVDLQDARALDGEAGSPAGPDALAADGQVPTAQLVAGWPARAAPVSLAGLATVRRAGQTPTTPGTSEGAPGRPQLLAPLSFQNPVYQMAAGLPLSPRGLGDSGSEGHSSLSSHSNSEELAAAAKLGSFSS;ATAAAAEDLGRRPGELARRQMSLTEKGGQPTVPRQNSAGPQRRGRTPPTLLSTLQYPRPSSGTLASASPDWAGPGARLRQQSSSSKGDSPELKPRAVHKQGPSPVSPNALDRTAAWLLTMNAQLLEDEALGPDPPHRDRLRSKEELSQAEKDLAVLQDKLRISTKKLEEYETLFKCQEETTQKLVLEYQARLEEGEERLRRQQEDKDIQMKGIISRLMSVEEELKKDHAEMQAAVDSKQKIIDAQEKRIASLDAANARLMSALTQLKERYSMQARNGISPTNPTKLQITENGEFRNSSNC.

16 residues IDQPPPPPPPPPPAPR MW 1668.9.

Functions as a scaffold protein implicated in the regulation of a large spectrum of both general and specialized signaling pathways.

>XP_005668832.1 PREDICTED: protein FAM171A2 [Sus scrofa] MPPPSGPSVLARLLPLLGLLLGGASRAPGKSPPEPPSPQEILIKVQVYVSGELVPLARASVDVFGNRMLLAAGTTDSEGVATLPLSYRLGTWVLVTAARPGFLTNSVPWRVDKLPLYASVSLYLLPERPATLILYEDLVHILLGSPGARSQPWVQFQRRAARLPVSSTYSQLWASLTPASTQQEMRAFPAFLGTDASSSGNGSWLELMPVAAVSVHLLAGNGTEVPLSGPIHLSLPVPSEPRALAVGTSIPAWRFDPKSGLWVRNGTGVIRKEGRQLYWTFISPQLGYWAAAMASPTSGLVTITSGIQDIGTYHTIFLLTILAALALLVLILLCLLIYYCRRRCLKPRQQHRKLQLSGPSDGNKRDQATSMSQLHLICGGPLEPAASGDPEAPPPGPLHSAFSSSRDLAASRDDFFRAKPRSASRPAAEPAGARGGEGAGLKGARSVEGPGGLEPGLEEYRRGPPGTATFLQEPPSPPPPFEHYLGHKGAAESKTPDFLLSQSVDQLERPPSLSQAGQLIFCGSIDHHSQVRHSYIDLQAGGGGRSTDASLDSGVDVHEARPARRRPLREERERAALALSEDTEPSSSESRTGLCSPEDNSLTPLLDEVAAPEGRAATVPRGRGRSRGDSSRSSASELRRDSLTSPEDELGAEVGDEAGDKKSPWQRREERPLMVFNVK.

17 residues AAAPPPPPPPPPPAPPR MW 1622.9.

>XP_005666867.1 PREDICTED: FH2 domain-containing protein 1 [Sus scrofa] MHVMNCVSSVSDKGNGNIAPAAGFMIGQTCPYSGAGFPPAPPPPPPPPLPGGPPVPPPPPGLPPPSHLNGYSHLGKKKRMRSFFWKTIPEEQVRGKTNIWTLAARQQHHYQIDTKTIEELFGQQEESAKSSPSRRGGPLNSSFREAREEITILDAKRSMNIGIFLKQFKKSPPSIVEDIHQGKSEHYGSETLREFLKLLPESEEIKKLKAFSGDVAKLSLADSFLHCLIQVPNYSLRIEAMVLKKEFLPSCSSLYTDMTILRTATKELMSCEELHSILHLVLQAGNIMNAGGYAGNAVGFKLSSLLKLADTKANKPGMNLLHFVAQEAQKKDAVLLNFSEKLHHVQEAARLSLDNTEAELHSLFVRTRSLKENIQRDGELCQQMEDFLQFAVEELSELERWKQELLAEAHTLIDFFCEDKDTVKLDECLQIFRDFCIKFNKAVKDNHDREVQELKQLQRLKEQEQKRRSWAAGELGFSRSSSENDVELLTKRGAEDPFLHSRPISPSHRPPNTRRSRLSLGASADRELLTFLESSTGNPEELKFNSLPRSCPRQAPPSRAWMESGEQRDQDSSQAHRLPASKDQEEATDPPSTWQSQLLAPRLEEPATALPRVRRSGVSILRKRNSEPLGLGPVRSPPLSPLALGIKEHELVTGLAQFDLQAPKGPEEPARLTMNDFSPMELMSVVGESPQAPRAPNDHRCEGLIPPCFSNEDLGNILLYVRAHAASRPYRESRAPSRSSFRKPSVKPLRNVPRPKPDEDKMCRSSSQGPESPEEAPRAPAAPSAPRGPAPVPSFARNTVASSSRCLRTDSPAVARPPGLTRTVSQRQLRAKGGPEEAAPKDGGALRRASSARGPRKGPELPEGPRAGSEASPKGRGAGERASVRLKDASRPALGKGLHPLRK.

14 residues PPPPSPPPPPPPPP MW 1366.7.

Formin-homology-domain-containing protein FHOD1 is involved in cell migration and adhesion, acting as a regulator of stress fibers organization, maturation of integrin-based adhesion sites, and podosome-associated contractility.

>XP_003127395.2 PREDICTED: proline-rich protein 12 isoform X1[Sus scrofa] MDRNYPSAGFGDPLGAGAGWSYERSAKASLVYGSSRTSHPETDILHRQAYAAPHPLQSYATNHHPAGLSGLFDTGLHHAGSAGPDASVMNLISALESRGPQPGPSASSLLSQFRSPSWQTAMHTPGPTELFISGALPGSSTFPSSSALSAYQHPASFGSRPFPVPSSLSLQDPPFSPPANGLLSPHDVLHLKPSQAPTVPSSLGFERLAGGGVLGPAGLGPAQTPPYRPGPPDPPPPPRHLPTQFNLLASSSAAAAAAAEQSSPQLYNFSGAAPGPPPPERALPRQDTVIKHYQRPASAQPPPPPPPAHALQHYLSCGGSYPSMGHRANLACSPLGGGEPSPGAGEPSKAGPSGATAGASGRAAGPEAAGGGGAGGGGGGYRPIIQSPGYKTGKGGYGAAAGGANRPPPPRSTATPKCQSLGGPAAAYATGKASGAGGAGGQAYSPGQPQGLLGPQAYGQGFGGGQAQDLSKGPSYSGGPQQPPNGPPPPGLATCQSYSPDQLQGQLYGVQGEPYPGPAAHSQGLPTASPSLSYSTGHSPALSGHGGGWGPSSLGGGGEASPSHIIRPLQSPPAPGRPPGVGSPGAPGKYLSSVLASAPFLAPPGAGSYAAGAGGYKGKGDGSELLAGPGGPPAERTEDEEFLIQHLLQAPSPPRTSGADGLVGEDGAADASKGLGGSGGAGGPPGTPYELAKEDPQRYHLQSVIRTSASLDEGATAALELGLGRLKEKKKGPERGGETPEGLATSVVHYGAGAKELGAFLQKSPPPPPPTAQSAQPTPHGLLLEAGGPDLPLVLPPPPPQLLPSVLSHAPSPSSSAPKVGVHLLEPAARDGAPPPPPPPPPPPMPLQLEAHLRSHGLEPGAPSPRLRPEESLEPPGAMQELLGALEPLPPGPGDTGVGPPTAEGKDPSGAYRSPSPQGTKAPRFVPLTSICFPDSLLQDEERSFFPTMEEMFGGGPADDYGKAGPPEDEGDPKAGAGPPPGPPAYDPYGPYCSSRASGAGPETPGLGLDPSKPPELPSTVNAEPLGLIQSGPHQAGGLTSPIFCSTKPKKLLKTSSFHLLRRRDPPFQTPKKLYAQEYEFEADEDKADVPADIRLNPRRLPDLVSSCRSRPALSPLGDIDFCPPNPGPDGPRRRGRKPTKAKRDGPPRPRGRPRIRPLEGPATAGPALASTPTDGAKKPRGRGRGRGRKAEEAGGTRLEPLKPLKIKLSVPKAGEGLGASSGEAVSGADPNSLDSSLTREKIEAKIKEVEEKQPEMKSGFMASFLDFLKSGKRHPPLYQAGLTPPLSPPKSVPPSVPARGLQPQPPSTPAVPHPPPAGAFGLGGALEAAESEGLGLGCPSPCKRLDEELKRNLETLPSFSSDEEDSVAKNRDLQESISSAISALDDPPLAGPKDTSTPDGPPLAADAAVPGPPPLPGLPSASSNGTPEPPLLEEKPPPSPPPVPTPQPPPPPPALPSPPPLVAPAPSSPPPQPPAPAPAPAPPALPSPPAPPPPPAAAAAPPPEEPAAPSPDDSEPPDARPLHLAKKQETAAVCGETDEEAGESGGEGIFRERDEFVIRAEDIPSLKLALQTGREPPPIWRVQKALLQKFTPEIKDGQRQFCATSNYLGYFGDAKNRYQRLYVKFLENVNKKDYVRVCARKPWHRPPVPVRRSGQAKGPSSSGGSSAPPPKAPAPPPKPETPDKMASEKPPEQTPETAVPEPPAPEKPSPPRLVEKEKEKERTPRGERPLRGERGTGGRQIRPDRGLTTGQPATSRLPKSRPTKVKAEPPPKKRKKWLKEAAGNASAGGGPPGSSSDSESSPGAPSEDERAVPGRLLKTRAMREMYRSYVEMLVSTALDPDMIQALEDTHDELYLPPMRKIDGLLNEHKKKVLKRLSLSPALQDALHTFPQLQVEQSGEGSPEEGAVRLRPAGEPYNRKTLSKLKRSVVRAQEFKVELDKSGYYTLYHSLHHYKYHTFLRCRDQTLAIEGGAEDLGQEEVVQQCMRNQPWLEQLFDSFSDLLAQAQAHSRCG.

19 residues APPPPPPPPPPPPPASEPK MW 1863.0.

20 residues LPPPPPPPPPPPPPPPPPPP MW 1975.1.

>NP_001231241.1 WAS/WASL-interacting protein family member2 Sus scrofa NQANTELPKLSRDEQRGRGALLQDICKGTKLKKVTNINDRSAPILEKPKGSSGGYGPGAAALQPKGGLFQGGVPKLRPVGAKDGSENLAGKPALQVPSSRAAAPRPPVSTASGRPQDDTDSNRASLPELPRTQRPSLPDLSRPHATSSTGMKHSSSAPPPPPPGRRANAPPTPLAMHSNKAPAYNREKPLPPTPGQRLHPGREGPSAPPPVKPPPSPVNIRTGPSGQSLAPPPPPYRQPPGVPNGPSSPTNESAPELPQRHNSLHRKTPGPVRGLAPPPPTSASPSLQSNRPPPPARDPPSRGAAPPPPPPMIRNGARDAPPPPPPYRMHGSEPLSRGKPPPPPSRTPAGPPPPPPPPLRNGHRDSITTVRSFLDDFESKYSFHPVEDFPAPEEYKHFQRVYPSKTNRAARGAPPLPPILR.

19 residues MPIPPPPPPPPGPPPPPTF MW 1926.0.

Plays an active role in the formation of cell surface protrusions downstream of activated PDGFB receptors. Plays an important role in actin-microspike formation through cooperation with WASL. May cooperate with WASP and WASL to induce mobilization and reorganization of the actin filament system.

>XP_005655053.1 PREDICTED: proline-rich protein 16 [Sus scrofa] MTDSSKTDTLNSSSSGTTASSIEKIKVQANAPLIKPPAHPSAILTVLRKLTPVKCEDPQRVVPTVNPVKTNGTLLRNGGFPGAPNKIPNGDICCKPGSIVDKAPVQPLMHRPEKDRCPQAGPRERVRFNEKVQYHGYCPDCDTRYNIKNREVHLHSEPVHPPGKLPPQGPHHPPPPHLPPFPLENGGLGISHSNSFPPLRPATVPPPTAPKPQKTILRKSTTTTV.

9 residue PNPPPPPPR MW 967.5.

Regulator of cell size that promotes cell size increase independently of mTOR and Hippo signaling pathways. Acts by stimulating the translation of specific mRNAs, including those encoding proteins affecting mitochondrial functions. Increases mitochondrial mass and respiration.

>XP_020955308.1 proline-rich membrane anchor 1 isoform X2 [Sus scrofa] MLLRDLVLRRGCCWPSLLLHCALHPLWGFVQVAHGEPQKSCSKVTDSCQHICQCRLLSAPAPNATSCPAEESWWSGLAIVIAVCCASLVFLTVLVIICYKAIKRKPLRKEENGTSVAEYPMTSSQSNKGVDVNSAVV.

16 residues PPPPLPPPPPPPPPPR MW 1645.9.

Required to anchor acetylcholinesterase (ACHE) to the basal lamina of the neuromuscular junction and to the membrane of neuronal synapses in brain. Also able to organize ACHE into tetramers.

## References

[1] Saxena A., Belinskaya T., Schopfer L.M., Lockridge O. (2018). Characterization of butyrylcholinesterase from porcine milk. Arch. Biochem Biophys..

[2] Heyndrickx G.V. (1963). Further investigations on the enzymes in human milk. Pediatrics.

[3] Walentin S., Levay G., Koranyi L., Endroczi E. (1988). Comparative analysis of enzyme activity in human colostrum, milk, and serum. Clin. Biochem..

[4] Brimijoin S., Tye S. (2017). Favorable impact on stress-related behaviors by modulating plasma butyrylcholinesterase. Cell. Mol. Neurobiol..

[5] Steculorum S.M., Collden G., Coupe B., Croizier S., Lockie S., Andrews Z.B., Jarosch F., Klussmann S., Bouret S.G. (2015). Neonatal ghrelin programs development of hypothalamic feeding circuits. J. Clin. Investig..

[6] Peng H., Schopfer L.M., Lockridge O. (2016). Origin of polyproline-rich peptides in human butyrylcholinesterase tetramers. Chem. Biol. Interact..

[7] Li H., Schopfer L.M., Masson P., Lockridge O. (2008). Lamellipodin proline rich peptides associated with native plasma butyrylcholinesterase tetramers. Biochem. J..

[8] Schopfer L.M., Delacour H., Masson P., Leroy J., Krejci E., Lockridge O. (2017). The C5 variant of the butyrylcholinesterase tetramer includes a noncovalently bound 60 kDa lamellipodin fragment. Molecules.

[9] Spieker J., Mudersbach T., Vogel-Hopker A., Layer P.G. (2017). Endochondral ossification is accelerated in cholinesterase-deficient mice and in avian mesenchymal micromass cultures. PLoS One.

[10] De la Hoz D., Doctor B.P., Ralston J.S., Rush R.S., Wolfe A.D. (1986). A simplified procedure for the purification of large quantities of fetal bovine serum acetylcholinesterase. Life Sci..

[11] Onder S., Schopfer L.M., Tacal O., Blake T.A., Johnson R.C., Lockridge O. (2018). Mass spectral detection of diethoxyphospho-tyrosine adducts on proteins from HEK293 cells using monoclonal antibody depY for enrichment. Chem. Res Toxicol..

