# Supplementary material for: Tetramer organizing polyproline-rich peptides identified by mass spectrometry after release of the peptides from Hupresin-purified butyrylcholinesterase tetramers isolated from milk of domestic pig (Sus scrofa)
Source: Data Brief. 2018 Aug 31;20:1607–19. doi: 10.1016/j.dib.2018.08.109 (PMC6157292; doi:10.1016/j.dib.2018.08.109)
Supplement: Supplementary file 1 — Supplementary material [file mmc1.docx]

**Declaration of interest**

The authors declare no competing financial interests.
